# Genetic Characterization of Highly Pathogenic Avian Influenza A(H5N6) Virus, Guangdong, China

**DOI:** 10.3201/eid2112.150809

**Published:** 2015-12

**Authors:** Chris Ka Pun Mok, Wen Da Guan, Xiao Qing Liu, Mart Matthias Lamers, Xiao Bo Li, Ming Wang, Tami Jing Shu Zhang, Qing Ling Zhang, Zheng Tu Li, Ji Cheng Huang, Jin Yan Lin, Yong Hui Zhang, Ping Zhao, Horace Hok Yeung Lee, Ling Chen, Yi Min Li, Joseph Sriyal Malik Peiris, Rong Chang Chen, Nan Shan Zhong, Zi Feng Yang

**Affiliations:** The University of Hong Kong,HKU–Pasteur Research Pole, Hong Kong, China (C.K.P Mok, M.M. Lamers, T.J.S. Zhang, H.H.Y. Lee, J.S.M. Peiris);; State Key Laboratory of Respiratory Disease, Guangzhou, China (W.D. Guan, X.Q. Liu, Q.L. Zhang, Z.T. Li, L. Chen, Y.M. Li, R.C. Chen, N.S. Zhong, Z.F. Yang);; Guangdong Inspection and Quarantine Technology Center, Guangzhou (X.B. Li, J.C. Huang);; Guangdong Center for Disease Control and Prevention, Guangzhou (J.Y. Lin, Y.H. Zhang);; Guangzhou Center for Disease Control and Prevention, Guangzhou (M. Wang);; Guangzhou Clifford Hospital, Guangzhou (P. Zhao)

**Keywords:** H5N6 subtype, influenza, avian, zoonoses, viruses, China

**To the Editor:** Since the first detection of the influenza A(H5N1) virus in geese in China during 1996 (http://www.cdc.gov/flu/avianflu/h5n1-virus.htm), H5 subtype viruses have continued to reassort and evolve, giving rise to multiple virus clades and gene constellations. Recently, clade 2.3.4.4 viruses have shown a predilection for genetic reassortment, giving rise to H5N2, H5N5, H5N6, and H5N8 virus subtypes, and have become globally widespread, causing infections in wild birds or poultry elsewhere in Asia, and in Europe and North America (*1*–*3*). The H5N6 subtype viruses have circulated in China since 2013 and have been mainly identified in ducks or chickens in the southern (Jiangxi, Guangdong) or western (Sichuan) areas (*4*,*5*). Two lineages of H5N6 viruses with distant genetic background were found among the H5N6 viruses isolated in China (*5*).

In China, there have been 3 cases of H5N6 virus infection among humans, causing 2 deaths. We recently reported the clinical characteristics and progression of a patient infected by the H5N6 virus in Guangzhou City, China, who was the second reported case-patient infected with this subtype (*6*). After having contact with poultry, he began to manifest an influenza-like illness on December 3, 2014, and progressed to a primary viral pneumonia. The H5N6 virus A/Guangzhou/39715/2014 (GenBank accession nos. KP765785–KP765792) was isolated from a throat swab specimen collected on day 8 of his illness by inoculation into 9–11-day-old, specific pathogen-free embryonated chicken eggs. He recovered from his infection and was discharged from the hospital on day 58.

Multiple sequence alignments showed that the hemagglutinin (HA) and neuraminidase (NA) genes of A/Guangzhou/39715/2014 shared the highest nucleotide identity with A/chicken/Dongguan/2690/2013 (H5N6) (99.4% and 98.3%, respectively) (Technical Appendix 1). All internal genes were also closely related to A/chicken/Dongguan/2690/2013 (H5N6), ranging from 98.5% nucleotide identity for the polymerase acidic (PA) gene and 100.0% for the matrix (M) gene. The genome segments were also 98.2%–99.7% identical to A/duck/Guangdong/GD012014 and 98.3%–99.4% identical to A/chicken/Laos/LPQ001/2014, which caused outbreaks in domestic ducks and poultry, respectively, indicating that these viruses have the same genotype.

HA gene phylogeny confirmed that this virus belonged to clade 2.3.4.4 (Technical Appendix 1, Figure 4). Notably, the HA genes of the H5N1, H5N2, and H5N8 viruses that were recently detected in wild birds in North America also belong to this clade, indicating that viruses from this clade are becoming globally widespread. More specifically, this isolate clustered within a sublineage that includes H5N6 isolates from poultry from Guangdong and Jiangxi provinces, China, and from Laos (*5*,*7*). The A/Sichuan/26221/2014 (H5N6) virus that recently caused a fatal human infection in Sichuan Province, China is also within clade 2.3.4.4, but clusters in a distinct sub-lineage (Figure, panel A).

**Figure:**
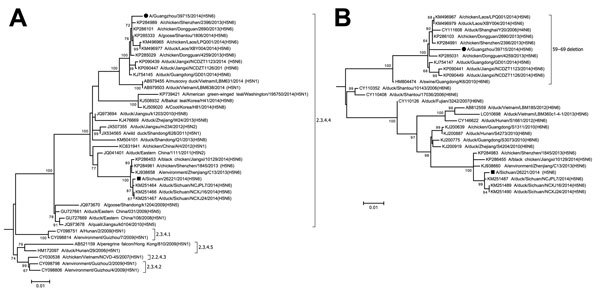
Phylogenetic trees of influenza A(H5N6) virus isolate A/Guangzhou/39715/2014 compared with other influenza viruses based on the A) hemagglutinin (HA) and B) neuraminidase (NA) genes, China. Maximum-likelihood trees were constructed by using the the general time reversible plus gamma distribution plus invariant sites (GTR+G+I) model in MEGA 6.06 (http://www.megasoftware.net). Bootstrap values were calculated on 1,000 replicates; only values >60% are shown. A/Guangzhou/39715/2014 and A/Sichuan/26221/2014 are indicated by a circle and a square, respectively. Brackets denote H5 subtype virus clades. Scale bars indicate nucleotide substitutions per site. Full HA and NA trees are provided in the Technical Appendix, Figures 1–8.

The HA cleavage site of both human isolates contained multiple basic amino acids, suggesting that that they are highly pathogenic avian influenza viruses. Amino acid substitutions E190D, Q226L, or G228S (H3 numbering) in the HA gene that are known to enhance binding to mammalian receptors were not found. The NA gene phylogeny showed that A/Guangzhou/39715/2014 is likely originated from group II lineage influenza A(H6N6) viruses that circulate among domestic ducks in China (*8*) (Figure, panel B). An 11-aa deletion at the residue 59–69 position of the NA protein was identified in the isolate of this study, in the other H5N6 viruses of the same cluster, and in an H4N6 virus isolate from a duck in Shanghai, China. This deletion was monophyletic and likely originated from A/swine/Guangdong/K6/2010 (H6N6)–like viruses (Figure, panel B). However, it was not observed in other 2.3.4.4 viruses, such as A/Sichuan/26221/2014.

No mutations associated with oseltamivir or amantadine resistance was found in NA or M2 genes. The internal genes of the current H5N6 isolate were similar to 2.3.2.1b H5N1 subtype viruses found in domestic ducks from south-central and eastern China (*5**,**7**–**10* [Technical Appendix 1]). The 6 internal genes are 97%–99% homologous to another isolate from a human, A/Sichuan/26221/2014, suggesting that the internal genes of the viruses may be reassorted from a common origin.

The phylogenetic clustering observed for the HA gene was also conserved for the internal genes. In contrast with all avian viruses within this clade, the current human isolate contains the mammalian adaptation mutation PB2-E627K, and A/Sichuan/26221/2014 has acquired PB2-D701N, suggesting a rapid acquisition of mammalian adaptation changes that likely arose after human infection.

There is still limited information on human disease caused by the emerging H5 lineage. Our genetic analysis suggests that the H5N6 virus isolated from the patient is originated from the avian host. Although the genetic background of H5N6 virus isolated from the third case in Yunnan Province, China, on January 2015 is still not known, the isolates from the human cases of H5N6 infection reported to date show distant genetic diversity, indicating that viruses from both clusters may pose a threat to humans. This rapidly evolving and globally spreading virus lineage thus provides a threat to global public health.

Technical Appendix 1 Full phylogenetic trees of polymerase basic 2 (PB2) (Technical Appendix Figure 1), PB1 (Technical Appendix Figure 2), polymerase acidic (PA) (Technical Appendix Figure 3), hemagglutinin (HA) (Technical Appendix Figure 4), nucleoprotein (NP) (Technical Appendix Figure 5), neuraminidase (NA) (Technical Appendix Figure 6), matrix (M) (Technical Appendix Figure 7), and nonstructural (NS) (Technical Appendix Figure 8) genes.

Technical Appendix 2 Authors and originating and submitting laboratories of the sequences from the Global Initiative on Sharing Avian Influenza Data EpiFlu Database, on which the current research is based.
