# Supplementary material for: Genetic Characterization of Highly Pathogenic Avian Influenza A(H5N6) Virus, Guangdong, China
Source: Emerg Infect Dis. 2015 Dec;21(12):2268–71. doi: 10.3201/eid2112.150809 (PMC4672456; doi:10.3201/eid2112.150809)
Supplement: Technical Appendix 1 — Full phylogenetic trees of polymerase basic 2 (PB2) (Technical Appendix Figure 1), PB1 (Technical Appendix Figure 2), polymerase acidic (PA) (Technical Appendix Figure 3), hemagglutinin (HA) (Technical Appendix Figure 4), nucleoprotein (NP) (Technical Appendix Figure 5), neuraminidase (NA) (Technical Appendix Figure 6), matrix (M) (Technical Appendix Figure 7), and nonstructural (NS) (Technical Appendix Figure 8) genes. [file 15-0809-Techapp-s1.pdf]

Article doi: http://dx.doi.org/10.3201/eid2112.150809

Genetic Characterization of Highly Pathogenic Avian Influenza A(H5N6) Virus, Guangdong, China

Online Technical Appendix 1

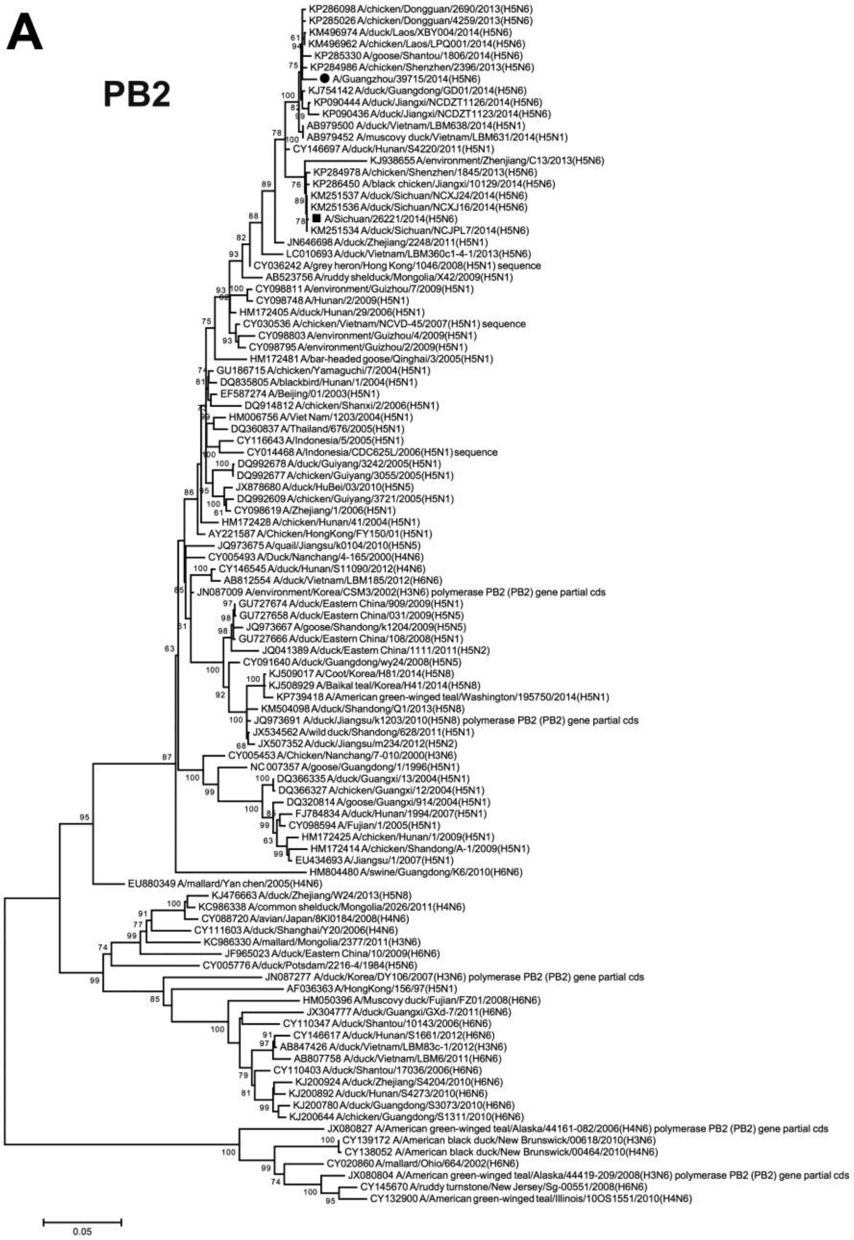

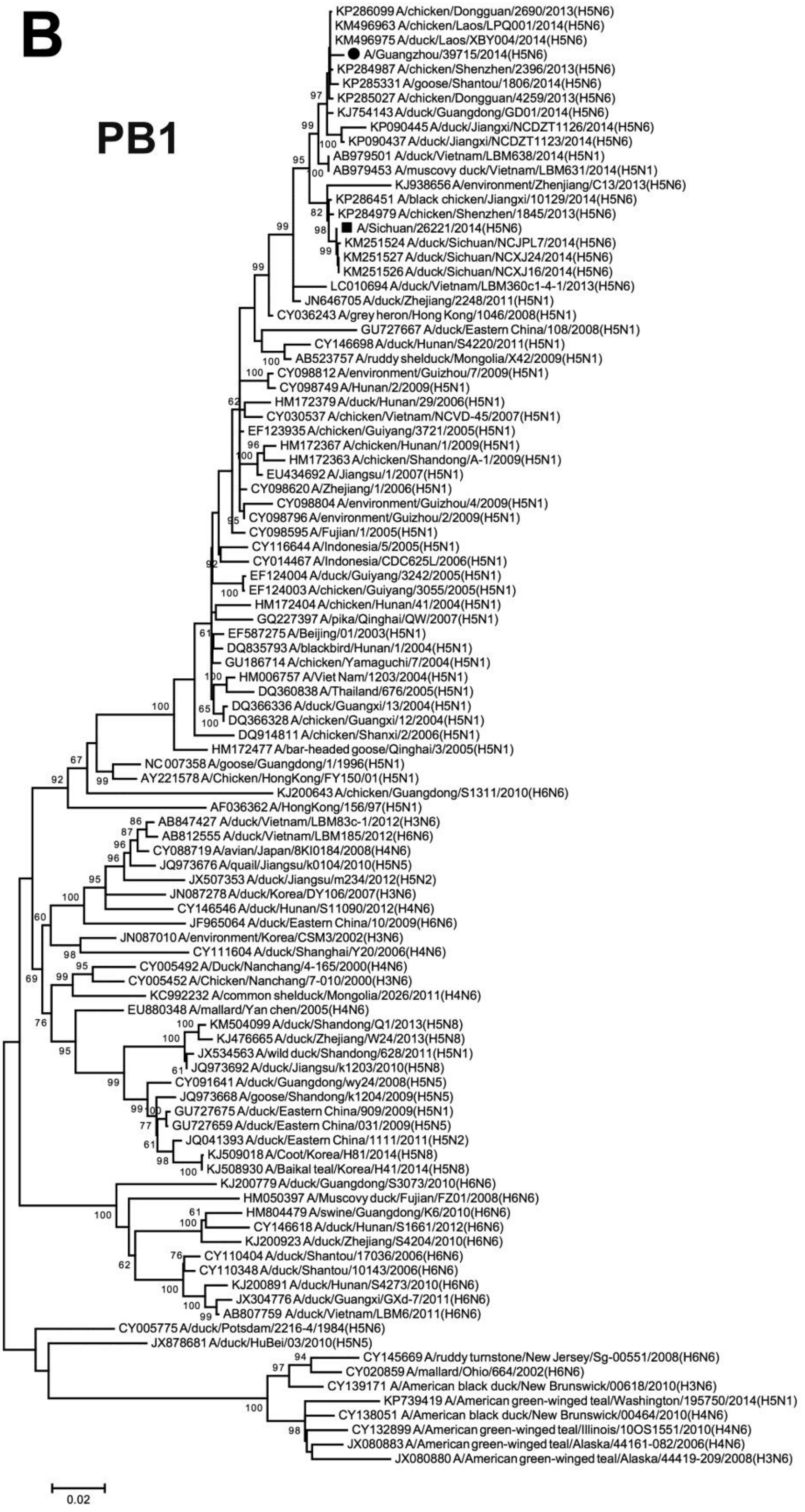
B
PB1
KP286099 A/chicken/Dongguan/2690/2013(H5N6)
KM496963 A/chicken/Laos/LPQ001/2014(H5N6)
KM496975 A/duck/Laos/XBY004/2014(H5N6)
A/Guangzhou/39715/2014(H5N6)
KP284987 A/chicken/Shenzhen/2396/2013(H5N6)
KP285331 A/goose/Shantou/1806/2014(H5N6)
KP285027 A/chicken/Dongguan/4259/2013(H5N6)
KJ754143 A/duck/Guangdong/GD01/2014(H5N6)
KP090445 A/duck/Jiangxi/NCDZT1126/2014(H5N6)
KP090437 A/duck/Jiangxi/NCDZT1123/2014(H5N6)
AB979501 A/duck/Vietnam/LBM638/2014(H5N1)
AB979453 A/muscovy duck/Vietnam/LBM631/2014(H5N1)
KJ938656 A/environment/Zhenjiang/C13/2013(H5N6)
KP286451 A/black chicken/Jiangxi/10129/2014(H5N6)
KP284979 A/chicken/Shenzhen/1845/2013(H5N6)
A/Sichuan/26221/2014(H5N6)
KM251524 A/duck/Sichuan/NCJPL7/2014(H5N6)
KM251527 A/duck/Sichuan/NCXJ24/2014(H5N6)
KM251526 A/duck/Sichuan/NCXJ16/2014(H5N6)
LC010694 A/duck/Vietnam/LBM360c1-4-1/2013(H5N6)
JN646705 A/duck/Zhejiang/2248/2011(H5N1)
CY036243 A/grey heron/Hong Kong/1046/2008(H5N1)
GU727667 A/duck/Eastern China/108/2008(H5N1)
CY146698 A/duck/Hunan/S4220/2011(H5N1)
AB523757 A/ruddy shelduck/Mongolia/X42/2009(H5N1)
CY098812 A/environment/Guizhou/7/2009(H5N1)
CY098749 A/Hunan/2/2009(H5N1)
HM172379 A/duck/Hunan/29/2006(H5N1)
CY030537 A/chicken/Vietnam/NCVD-45/2007(H5N1)
EF123935 A/chicken/Guiyang/3721/2005(H5N1)
HM172367 A/chicken/Hunan/1/2009(H5N1)
HM172363 A/chicken/Shandong/A-1/2009(H5N1)
EU434692 A/Jiangsu/1/2007(H5N1)
CY098620 A/Zhejiang/1/2006(H5N1)
CY098804 A/environment/Guizhou/4/2009(H5N1)
CY098796 A/environment/Guizhou/2/2009(H5N1)
CY098595 A/Fujian/1/2005(H5N1)
CY116644 A/Indonesia/5/2005(H5N1)
CY014467 A/Indonesia/CDC625L/2006(H5N1)
EF124004 A/duck/Guiyang/3242/2005(H5N1)
EF124003 A/chicken/Guiyang/3055/2005(H5N1)
HM172404 A/chicken/Hunan/41/2004(H5N1)
GQ227397 A/pika/Qinghai/QW/2007(H5N1)
EF587275 A/Beijing/01/2003(H5N1)
DQ835793 A/blackbird/Hunan/1/2004(H5N1)
GU186714 A/chicken/Yamaguchi/7/2004(H5N1)
HM006757 A/Viet Nam/1203/2004(H5N1)
DQ360838 A/Thailand/676/2005(H5N1)
DQ366336 A/duck/Guangxi/13/2004(H5N1)
DQ366328 A/chicken/Guangxi/12/2004(H5N1)
DQ914811 A/chicken/Shanxi/2/2006(H5N1)
HM172477 A/bar-headed goose/Qinghai/3/2005(H5N1)
NC 007358 A/goose/Guangdong/1/1996(H5N1)
AY221578 A/Chicken/HongKong/FY150/01(H5N1)
KJ200643 A/chicken/Guangdong/S1311/2010(H6N6)
AF036362 A/HongKong/156/97(H5N1)
AB847427 A/duck/Vietnam/LBM83c-1/2012(H3N6)
AB812555 A/duck/Vietnam/LBM185/2012(H6N6)
CY088719 A/avian/Japan/8KI0184/2008(H4N6)
JQ973676 A/quail/Jiangsu/k0104/2010(H5N5)
JX507353 A/duck/Jiangsu/m234/2012(H5N2)
JN087278 A/duck/Korea/DY106/2007(H3N6)
CY146546 A/duck/Hunan/S11090/2012(H4N6)
JF965064 A/duck/Eastern China/10/2009(H6N6)
JN087010 A/environment/Korea/CSM3/2002(H3N6)
CY111604 A/duck/Shanghai/Y20/2006(H4N6)
CY005492 A/Duck/Nanchang/4-165/2000(H4N6)
CY005452 A/Chicken/Nanchang/7-010/2000(H3N6)
KC992232 A/common shelduck/Mongolia/2026/2011(H4N6)
EU880348 A/mallard/Yan chen/2005(H4N6)
KM504099 A/duck/Shandong/Q1/2013(H5N8)
KJ476665 A/duck/Zhejiang/W24/2013(H5N8)
JX534563 A/wild duck/Shandong/628/2011(H5N1)
JQ973692 A/duck/Jiangsu/k1203/2010(H5N8)
CY091641 A/duck/Guangdong/wy24/2008(H5N5)
JQ973668 A/goose/Shandong/k1204/2009(H5N5)
GU727675 A/duck/Eastern China/909/2009(H5N1)
GU727659 A/duck/Eastern China/031/2009(H5N5)
JQ041393 A/duck/Eastern China/1111/2011(H5N2)
KJ509018 A/Coot/Korea/H81/2014(H5N8)
KJ508930 A/Baikal teal/Korea/H41/2014(H5N8)
KJ200779 A/duck/Guangdong/S3073/2010(H6N6)
HM050397 A/Muscovy duck/Fujian/FZ01/2008(H6N6)
HM804479 A/swine/Guangdong/K6/2010(H6N6)
CY146618 A/duck/Hunan/S1661/2012(H6N6)
KJ200923 A/duck/Zhejiang/S4204/2010(H6N6)
CY110404 A/duck/Shantou/17036/2006(H6N6)
CY110348 A/duck/Shantou/10143/2006(H6N6)
KJ200891 A/duck/Hunan/S4273/2010(H6N6)
JX304776 A/duck/Guangxi/GXd-7/2011(H6N6)
AB807759 A/duck/Vietnam/LBM6/2011(H6N6)
CY005775 A/duck/Potsdam/2216-4/1984(H5N6)
JX878681 A/duck/HuBei/03/2010(H5N5)
CY145669 A/ruddy turnstone/New Jersey/Sg-00551/2008(H6N6)
CY020859 A/mallard/Ohio/664/2002(H6N6)
CY139171 A/American black duck/New Brunswick/00618/2010(H3N6)
KP739419 A/American green-winged teal/Washington/195750/2014(H5N1)
CY138051 A/American black duck/New Brunswick/00464/2010(H4N6)
CY132899 A/American green-winged teal/Illinois/10OS1551/2010(H4N6)
JX080883 A/American green-winged teal/Alaska/44161-082/2006(H4N6)
JX080880 A/American green-winged teal/Alaska/44419-209/2008(H3N6)
0.02

C

PA

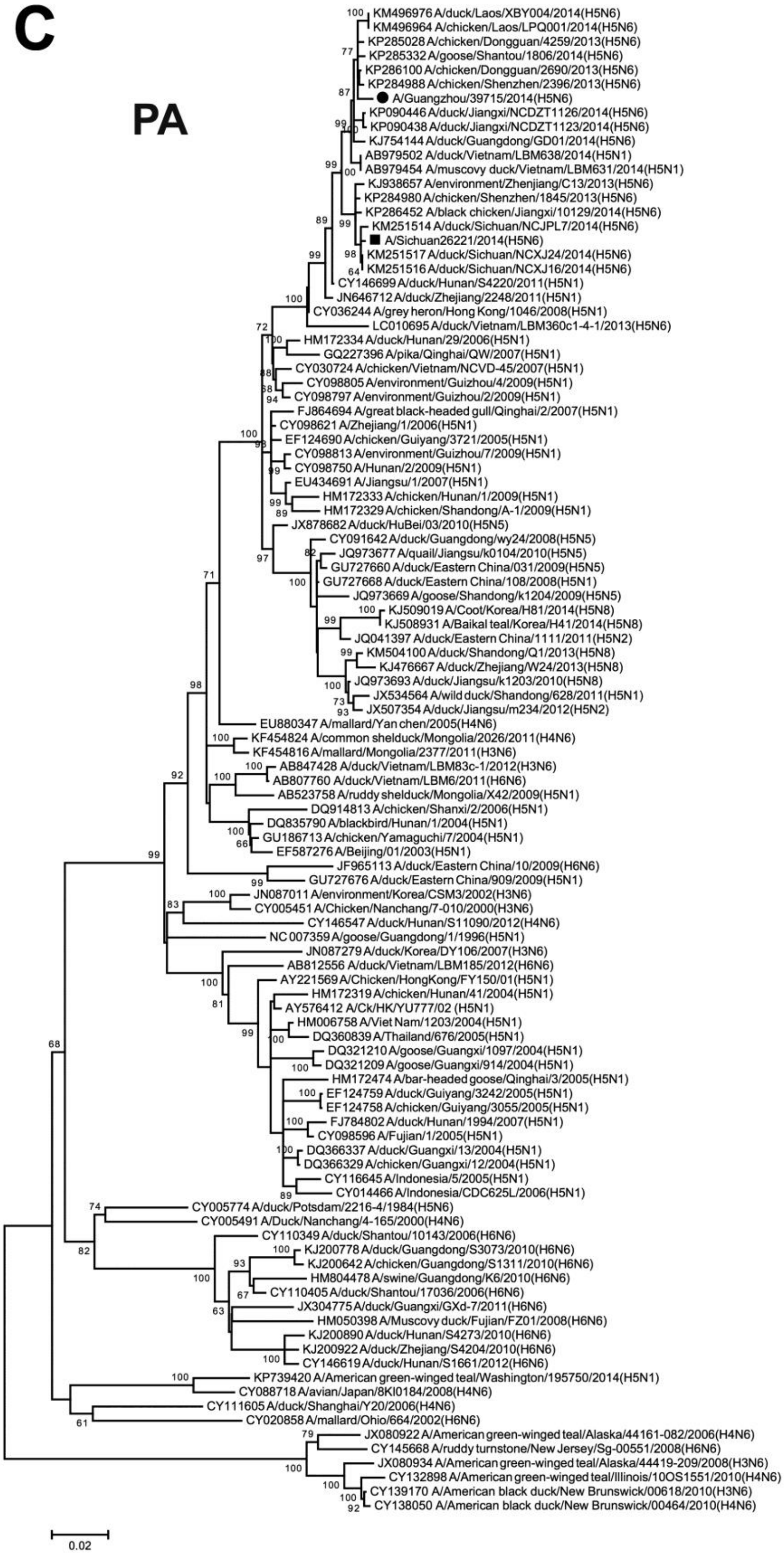

KM496976 A/duck/Laos/XBY004/2014(H5N6)
KM496964 A/chicken/Laos/LPQ001/2014(H5N6)
KP285028 A/chicken/Dongguan/4259/2013(H5N6)
KP285332 A/goose/Shantou/1806/2014(H5N6)
KP286100 A/chicken/Dongguan/2690/2013(H5N6)
KP284988 A/chicken/Shenzhen/2396/2013(H5N6)
A/Guangzhou/39715/2014(H5N6)
KP090446 A/duck/Jiangxi/NCDZT1126/2014(H5N6)
KP090438 A/duck/Jiangxi/NCDZT1123/2014(H5N6)
KJ754144 A/duck/Guangdong/GD01/2014(H5N6)
AB979502 A/duck/Vietnam/LBM638/2014(H5N1)
AB979454 A/muscovy duck/Vietnam/LBM631/2014(H5N1)
KJ938657 A/environment/Zhenjiang/C13/2013(H5N6)
KP284980 A/chicken/Shenzhen/1845/2013(H5N6)
KP286452 A/black chicken/Jiangxi/10129/2014(H5N6)
KM251514 A/duck/Sichuan/NCJPL7/2014(H5N6)
A/Sichuan26221/2014(H5N6)
KM251517 A/duck/Sichuan/NCXJ24/2014(H5N6)
KM251516 A/duck/Sichuan/NCXJ16/2014(H5N6)
CY146699 A/duck/Hunan/S4220/2011(H5N1)
JN646712 A/duck/Zhejiang/2248/2011(H5N1)
CY036244 A/grey heron/Hong Kong/1046/2008(H5N1)
LC010695 A/duck/Vietnam/LBM360c1-4-1/2013(H5N6)
HM172334 A/duck/Hunan/29/2006(H5N1)
GQ227396 A/pika/Qinghai/QW/2007(H5N1)
CY030724 A/chicken/Vietnam/NCVD-45/2007(H5N1)
CY098805 A/environment/Guizhou/4/2009(H5N1)
CY098797 A/environment/Guizhou/2/2009(H5N1)
FJ864694 A/great black-headed gull/Qinghai/2/2007(H5N1)
CY098621 A/Zhejiang/1/2006(H5N1)
EF124690 A/chicken/Guiyang/3721/2005(H5N1)
CY098813 A/environment/Guizhou/7/2009(H5N1)
CY098750 A/Hunan/2/2009(H5N1)
EU434691 A/Jiangsu/1/2007(H5N1)
HM172333 A/chicken/Hunan/1/2009(H5N1)
HM172329 A/chicken/Shandong/A-1/2009(H5N1)
JX878682 A/duck/HuBei/03/2010(H5N5)
CY091642 A/duck/Guangdong/wy24/2008(H5N5)
JQ973677 A/quail/Jiangsu/k0104/2010(H5N5)
GU727660 A/duck/Eastern China/031/2009(H5N5)
GU727668 A/duck/Eastern China/108/2008(H5N1)
JQ973669 A/goose/Shandong/k1204/2009(H5N5)
KJ509019 A/Coot/Korea/H81/2014(H5N8)
KJ508931 A/Baikal teal/Korea/H41/2014(H5N8)
JQ041397 A/duck/Eastern China/1111/2011(H5N2)
KM504100 A/duck/Shandong/Q1/2013(H5N8)
KJ476667 A/duck/Zhejiang/W24/2013(H5N8)
JQ973693 A/duck/Jiangsu/k1203/2010(H5N8)
JX534564 A/wild duck/Shandong/628/2011(H5N1)
JX507354 A/duck/Jiangsu/m234/2012(H5N2)
EU880347 A/mallard/Yan chen/2005(H4N6)
KF454824 A/common shelduck/Mongolia/2026/2011(H4N6)
KF454816 A/mallard/Mongolia/2377/2011(H3N6)
AB847428 A/duck/Vietnam/LBM83c-1/2012(H3N6)
AB807760 A/duck/Vietnam/LBM6/2011(H6N6)
AB523758 A/ruddy shelduck/Mongolia/X42/2009(H5N1)
DQ914813 A/chicken/Shanxi/2/2006(H5N1)
DQ835790 A/blackbird/Hunan/1/2004(H5N1)
GU186713 A/chicken/Yamaguchi/7/2004(H5N1)
EF587276 A/Beijing/01/2003(H5N1)
JF965113 A/duck/Eastern China/10/2009(H6N6)
GU727676 A/duck/Eastern China/909/2009(H5N1)
JN087011 A/environment/Korea/CSM3/2002(H3N6)
CY005451 A/Chicken/Nanchang/7-010/2000(H3N6)
CY146547 A/duck/Hunan/S11090/2012(H4N6)
NC 007359 A/goose/Guangdong/1/1996(H5N1)
JN087279 A/duck/Korea/DY106/2007(H3N6)
AB812556 A/duck/Vietnam/LBM185/2012(H6N6)
AY221569 A/Chicken/HongKong/FY150/01(H5N1)
HM172319 A/chicken/Hunan/41/2004(H5N1)
AY576412 A/Ck/HK/YU777/02 (H5N1)
HM006758 A/Viet Nam/1203/2004(H5N1)
DQ360839 A/Thailand/676/2005(H5N1)
DQ321210 A/goose/Guangxi/1097/2004(H5N1)
DQ321209 A/goose/Guangxi/914/2004(H5N1)
HM172474 A/bar-headed goose/Qinghai/3/2005(H5N1)
EF124759 A/duck/Guiyang/3242/2005(H5N1)
EF124758 A/chicken/Guiyang/3055/2005(H5N1)
FJ784802 A/duck/Hunan/1994/2007(H5N1)
CY098596 A/Fujian/1/2005(H5N1)
DQ366337 A/duck/Guangxi/13/2004(H5N1)
DQ366329 A/chicken/Guangxi/12/2004(H5N1)
CY116645 A/Indonesia/5/2005(H5N1)
CY014466 A/Indonesia/CDC625L/2006(H5N1)
CY005774 A/duck/Potsdam/2216-4/1984(H5N6)
CY005491 A/Duck/Nanchang/4-165/2000(H4N6)
CY110349 A/duck/Shantou/10143/2006(H6N6)
KJ200778 A/duck/Guangdong/S3073/2010(H6N6)
KJ200642 A/chicken/Guangdong/S1311/2010(H6N6)
HM804478 A/swine/Guangdong/K6/2010(H6N6)
CY110405 A/duck/Shantou/17036/2006(H6N6)
JX304775 A/duck/Guangxi/GXd-7/2011(H6N6)
HM050398 A/Muscovy duck/Fujian/FZ01/2008(H6N6)
KJ200890 A/duck/Hunan/S4273/2010(H6N6)
KJ200922 A/duck/Zhejiang/S4204/2010(H6N6)
CY146619 A/duck/Hunan/S1661/2012(H6N6)
KP739420 A/American green-winged teal/Washington/195750/2014(H5N1)
CY088718 A/avian/Japan/8KI0184/2008(H4N6)
CY111605 A/duck/Shanghai/Y20/2006(H4N6)
CY020858 A/mallard/Ohio/664/2002(H6N6)
JX080922 A/American green-winged teal/Alaska/44161-082/2006(H4N6)
CY145668 A/ruddy turnstone/New Jersey/Sg-00551/2008(H6N6)
JX080934 A/American green-winged teal/Alaska/44419-209/2008(H3N6)
CY132898 A/American green-winged teal/Illinois/10OS1551/2010(H4N6)
CY139170 A/American black duck/New Brunswick/00618/2010(H3N6)
CY138050 A/American black duck/New Brunswick/00464/2010(H4N6)
0.02

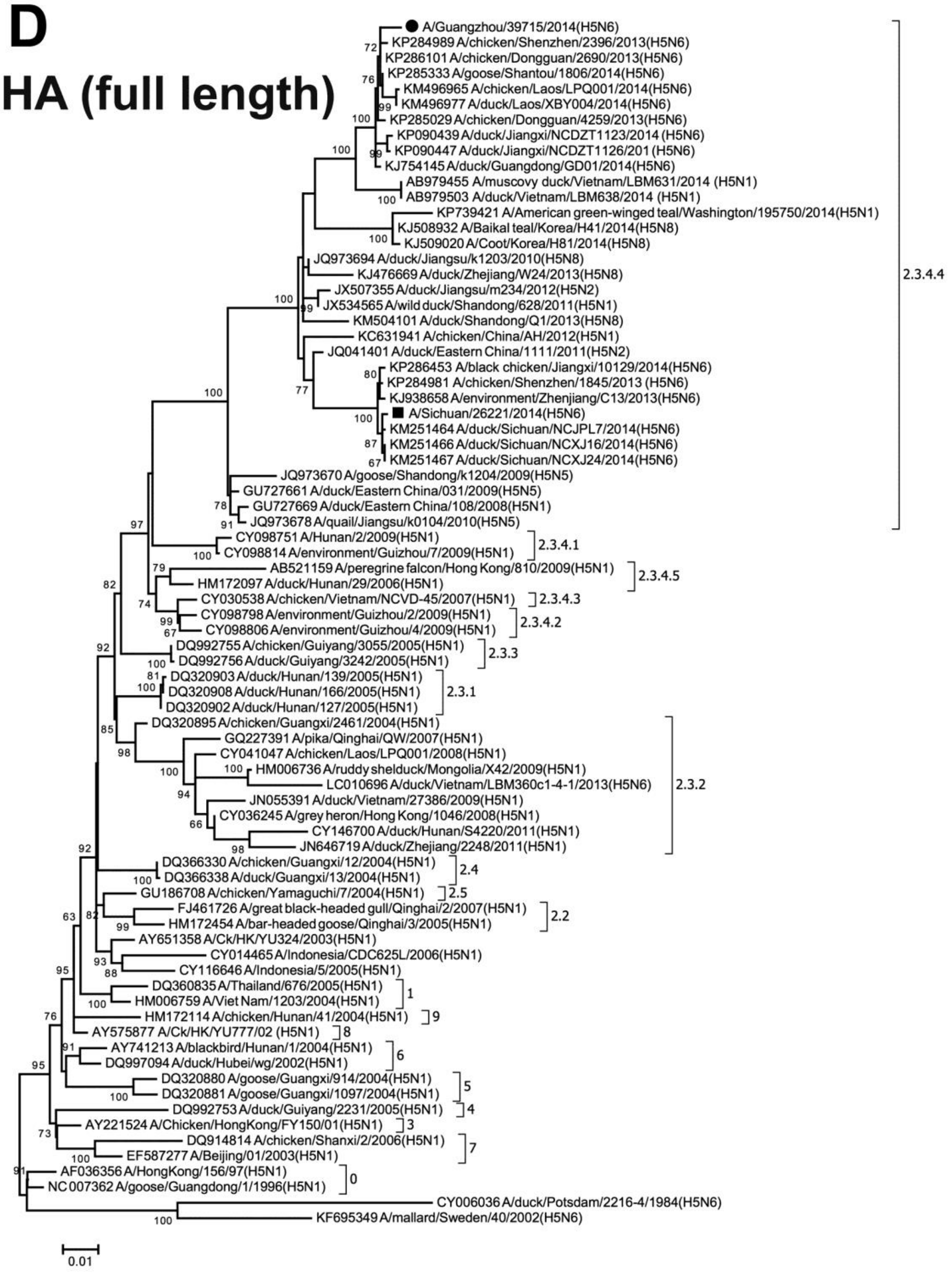
D
HA (full length)
A/Guangzhou/39715/2014(H5N6)
KP284989 A/chicken/Shenzhen/2396/2013(H5N6)
KP286101 A/chicken/Dongguan/2690/2013(H5N6)
KP285333 A/goose/Shantou/1806/2014(H5N6)
KM496965 A/chicken/Laos/LPQ001/2014(H5N6)
KM496977 A/duck/Laos/XBY004/2014(H5N6)
KP285029 A/chicken/Dongguan/4259/2013(H5N6)
KP090439 A/duck/Jiangxi/NCDZT1123/2014 (H5N6)
KP090447 A/duck/Jiangxi/NCDZT1126/201 (H5N6)
KJ754145 A/duck/Guangdong/GD01/2014(H5N6)
AB979455 A/muscovy duck/Vietnam/LBM631/2014 (H5N1)
AB979503 A/duck/Vietnam/LBM638/2014 (H5N1)
KP739421 A/American green-winged teal/Washington/195750/2014(H5N1)
KJ508932 A/Baikal teal/Korea/H41/2014(H5N8)
KJ509020 A/Coot/Korea/H81/2014(H5N8)
JQ973694 A/duck/Jiangsu/k1203/2010(H5N8)
KJ476669 A/duck/Zhejiang/W24/2013(H5N8)
JX507355 A/duck/Jiangsu/m234/2012(H5N2)
JX534565 A/wild duck/Shandong/628/2011(H5N1)
KM504101 A/duck/Shandong/Q1/2013(H5N8)
KC631941 A/chicken/China/AH/2012(H5N1)
JQ041401 A/duck/Eastern China/1111/2011(H5N2)
KP286453 A/black chicken/Jiangxi/10129/2014(H5N6)
KP284981 A/chicken/Shenzhen/1845/2013 (H5N6)
KJ938658 A/environment/Zhenjiang/C13/2013(H5N6)
A/Sichuan/26221/2014(H5N6)
KM251464 A/duck/Sichuan/NCJPL7/2014(H5N6)
KM251466 A/duck/Sichuan/NCXJ16/2014(H5N6)
KM251467 A/duck/Sichuan/NCXJ24/2014(H5N6)
JQ973670 A/goose/Shandong/k1204/2009(H5N5)
GU727661 A/duck/Eastern China/031/2009(H5N5)
GU727669 A/duck/Eastern China/108/2008(H5N1)
JQ973678 A/quail/Jiangsu/k0104/2010(H5N5)
2.3.4.4
CY098751 A/Hunan/2/2009(H5N1)
CY098814 A/environment/Guizhou/7/2009(H5N1)
2.3.4.1
AB521159 A/peregrine falcon/Hong Kong/810/2009(H5N1)
HM172097 A/duck/Hunan/29/2006(H5N1)
2.3.4.5
CY030538 A/chicken/Vietnam/NCVD-45/2007(H5N1)
2.3.4.3
CY098798 A/environment/Guizhou/2/2009(H5N1)
CY098806 A/environment/Guizhou/4/2009(H5N1)
2.3.4.2
DQ992755 A/chicken/Guiyang/3055/2005(H5N1)
DQ992756 A/duck/Guiyang/3242/2005(H5N1)
2.3.3
DQ320903 A/duck/Hunan/139/2005(H5N1)
DQ320908 A/duck/Hunan/166/2005(H5N1)
DQ320902 A/duck/Hunan/127/2005(H5N1)
2.3.1
DQ320895 A/chicken/Guangxi/2461/2004(H5N1)
GQ227391 A/pika/Qinghai/QW/2007(H5N1)
CY041047 A/chicken/Laos/LPQ001/2008(H5N1)
HM006736 A/ruddy shelduck/Mongolia/X42/2009(H5N1)
LC010696 A/duck/Vietnam/LBM360c1-4-1/2013(H5N6)
JN055391 A/duck/Vietnam/27386/2009(H5N1)
CY036245 A/grey heron/Hong Kong/1046/2008(H5N1)
CY146700 A/duck/Hunan/S4220/2011(H5N1)
JN646719 A/duck/Zhejiang/2248/2011(H5N1)
2.3.2
DQ366330 A/chicken/Guangxi/12/2004(H5N1)
DQ366338 A/duck/Guangxi/13/2004(H5N1)
2.4
GU186708 A/chicken/Yamaguchi/7/2004(H5N1)
2.5
FJ461726 A/great black-headed gull/Qinghai/2/2007(H5N1)
HM172454 A/bar-headed goose/Qinghai/3/2005(H5N1)
2.2
AY651358 A/Ck/HK/YU324/2003(H5N1)
CY014465 A/Indonesia/CDC625L/2006(H5N1)
CY116646 A/Indonesia/5/2005(H5N1)
DQ360835 A/Thailand/676/2005(H5N1)
HM006759 A/Viet Nam/1203/2004(H5N1)
1
HM172114 A/chicken/Hunan/41/2004(H5N1)
9
AY575877 A/Ck/HK/YU777/02 (H5N1)
8
AY741213 A/blackbird/Hunan/1/2004(H5N1)
DQ997094 A/duck/Hubei/wg/2002(H5N1)
6
DQ320880 A/goose/Guangxi/914/2004(H5N1)
DQ320881 A/goose/Guangxi/1097/2004(H5N1)
5
DQ992753 A/duck/Guiyang/2231/2005(H5N1)
4
AY221524 A/Chicken/HongKong/FY150/01(H5N1)
3
DQ914814 A/chicken/Shanxi/2/2006(H5N1)
EF587277 A/Beijing/01/2003(H5N1)
7
AF036356 A/HongKong/156/97(H5N1)
NC 007362 A/goose/Guangdong/1/1996(H5N1)
0
CY006036 A/duck/Potsdam/2216-4/1984(H5N6)
KF695349 A/mallard/Sweden/40/2002(H5N6)
0.01

E

NP

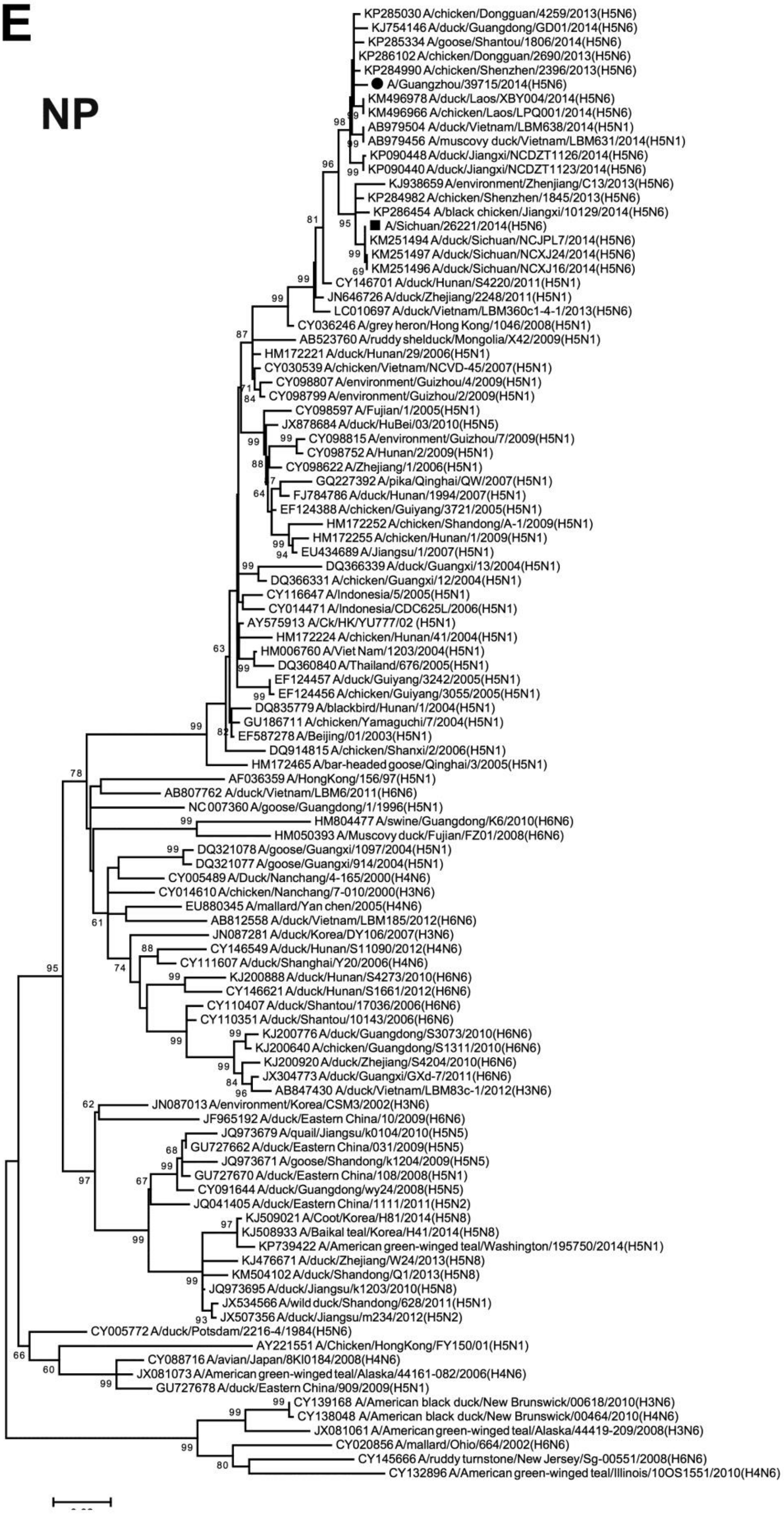
KP285030 A/chicken/Dongguan/4259/2013(H5N6)
KJ754146 A/duck/Guangdong/GD01/2014(H5N6)
KP285334 A/goose/Shantou/1806/2014(H5N6)
KP286102 A/chicken/Dongguan/2690/2013(H5N6)
KP284990 A/chicken/Shenzhen/2396/2013(H5N6)
A/Guangzhou/39715/2014(H5N6)
KM496978 A/duck/Laos/XBY004/2014(H5N6)
KM496966 A/chicken/Laos/LPQ001/2014(H5N6)
AB979504 A/duck/Vietnam/LBM638/2014(H5N1)
AB979456 A/muscovy duck/Vietnam/LBM631/2014(H5N1)
KP090448 A/duck/Jiangxi/NCDZT1126/2014(H5N6)
KP090440 A/duck/Jiangxi/NCDZT1123/2014(H5N6)
KJ938659 A/environment/Zhenjiang/C13/2013(H5N6)
KP284982 A/chicken/Shenzhen/1845/2013(H5N6)
KP286454 A/black chicken/Jiangxi/10129/2014(H5N6)
A/Sichuan/26221/2014(H5N6)
KM251494 A/duck/Sichuan/NCJPL7/2014(H5N6)
KM251497 A/duck/Sichuan/NCXJ24/2014(H5N6)
KM251496 A/duck/Sichuan/NCXJ16/2014(H5N6)
CY146701 A/duck/Hunan/S4220/2011(H5N1)
JN646726 A/duck/Zhejiang/2248/2011(H5N1)
LC010697 A/duck/Vietnam/LBM360c1-4-1/2013(H5N6)
CY036246 A/grey heron/Hong Kong/1046/2008(H5N1)
AB523760 A/ruddy shelduck/Mongolia/X42/2009(H5N1)
HM172221 A/duck/Hunan/29/2006(H5N1)
CY030539 A/chicken/Vietnam/NCVD-45/2007(H5N1)
CY098807 A/environment/Guizhou/4/2009(H5N1)
CY098799 A/environment/Guizhou/2/2009(H5N1)
CY098597 A/Fujian/1/2005(H5N1)
JX878684 A/duck/HuBei/03/2010(H5N5)
CY098815 A/environment/Guizhou/7/2009(H5N1)
CY098752 A/Hunan/2/2009(H5N1)
CY098622 A/Zhejiang/1/2006(H5N1)
GQ227392 A/pika/Qinghai/QW/2007(H5N1)
FJ784786 A/duck/Hunan/1994/2007(H5N1)
EF124388 A/chicken/Guiyang/3721/2005(H5N1)
HM172252 A/chicken/Shandong/A-1/2009(H5N1)
HM172255 A/chicken/Hunan/1/2009(H5N1)
EU434689 A/Jiangsu/1/2007(H5N1)
DQ366339 A/duck/Guangxi/13/2004(H5N1)
DQ366331 A/chicken/Guangxi/12/2004(H5N1)
CY116647 A/Indonesia/5/2005(H5N1)
CY014471 A/Indonesia/CDC625L/2006(H5N1)
AY575913 A/Ck/HK/YU777/02 (H5N1)
HM172224 A/chicken/Hunan/41/2004(H5N1)
HM006760 A/Viet Nam/1203/2004(H5N1)
DQ360840 A/Thailand/676/2005(H5N1)
EF124457 A/duck/Guiyang/3242/2005(H5N1)
EF124456 A/chicken/Guiyang/3055/2005(H5N1)
DQ835779 A/blackbird/Hunan/1/2004(H5N1)
GU186711 A/chicken/Yamaguchi/7/2004(H5N1)
EF587278 A/Beijing/01/2003(H5N1)
DQ914815 A/chicken/Shanxi/2/2006(H5N1)
HM172465 A/bar-headed goose/Qinghai/3/2005(H5N1)
AF036359 A/HongKong/156/97(H5N1)
AB807762 A/duck/Vietnam/LBM6/2011(H6N6)
NC 007360 A/goose/Guangdong/1/1996(H5N1)
HM804477 A/swine/Guangdong/K6/2010(H6N6)
HM050393 A/Muscovy duck/Fujian/FZ01/2008(H6N6)
DQ321078 A/goose/Guangxi/1097/2004(H5N1)
DQ321077 A/goose/Guangxi/914/2004(H5N1)
CY005489 A/Duck/Nanchang/4-165/2000(H4N6)
CY014610 A/chicken/Nanchang/7-010/2000(H3N6)
EU880345 A/mallard/Yan chen/2005(H4N6)
AB812558 A/duck/Vietnam/LBM185/2012(H6N6)
JN087281 A/duck/Korea/DY106/2007(H3N6)
CY146549 A/duck/Hunan/S11090/2012(H4N6)
CY111607 A/duck/Shanghai/Y20/2006(H4N6)
KJ200888 A/duck/Hunan/S4273/2010(H6N6)
CY146621 A/duck/Hunan/S1661/2012(H6N6)
CY110407 A/duck/Shantou/17036/2006(H6N6)
CY110351 A/duck/Shantou/10143/2006(H6N6)
KJ200776 A/duck/Guangdong/S3073/2010(H6N6)
KJ200640 A/chicken/Guangdong/S1311/2010(H6N6)
KJ200920 A/duck/Zhejiang/S4204/2010(H6N6)
JX304773 A/duck/Guangxi/GXd-7/2011(H6N6)
AB847430 A/duck/Vietnam/LBM83c-1/2012(H3N6)
JN087013 A/environment/Korea/CSM3/2002(H3N6)
JF965192 A/duck/Eastern China/10/2009(H6N6)
JQ973679 A/quail/Jiangsu/k0104/2010(H5N5)
GU727662 A/duck/Eastern China/031/2009(H5N5)
JQ973671 A/goose/Shandong/k1204/2009(H5N5)
GU727670 A/duck/Eastern China/108/2008(H5N1)
CY091644 A/duck/Guangdong/wy24/2008(H5N5)
JQ041405 A/duck/Eastern China/1111/2011(H5N2)
KJ509021 A/Coot/Korea/H81/2014(H5N8)
KJ508933 A/Baikal teal/Korea/H41/2014(H5N8)
KP739422 A/American green-winged teal/Washington/195750/2014(H5N1)
KJ476671 A/duck/Zhejiang/W24/2013(H5N8)
KM504102 A/duck/Shandong/Q1/2013(H5N8)
JQ973695 A/duck/Jiangsu/k1203/2010(H5N8)
JX534566 A/wild duck/Shandong/628/2011(H5N1)
JX507356 A/duck/Jiangsu/m234/2012(H5N2)
CY005772 A/duck/Potsdam/2216-4/1984(H5N6)
AY221551 A/Chicken/HongKong/FY150/01(H5N1)
CY088716 A/avian/Japan/8KI0184/2008(H4N6)
JX081073 A/American green-winged teal/Alaska/44161-082/2006(H4N6)
GU727678 A/duck/Eastern China/909/2009(H5N1)
CY139168 A/American black duck/New Brunswick/00618/2010(H3N6)
CY138048 A/American black duck/New Brunswick/00464/2010(H4N6)
JX081061 A/American green-winged teal/Alaska/44419-209/2008(H3N6)
CY020856 A/mallard/Ohio/664/2002(H6N6)
CY145666 A/ruddy turnstone/New Jersey/Sg-00551/2008(H6N6)
CY132896 A/American green-winged teal/Illinois/10OS1551/2010(H4N6)

F

NA (full length)

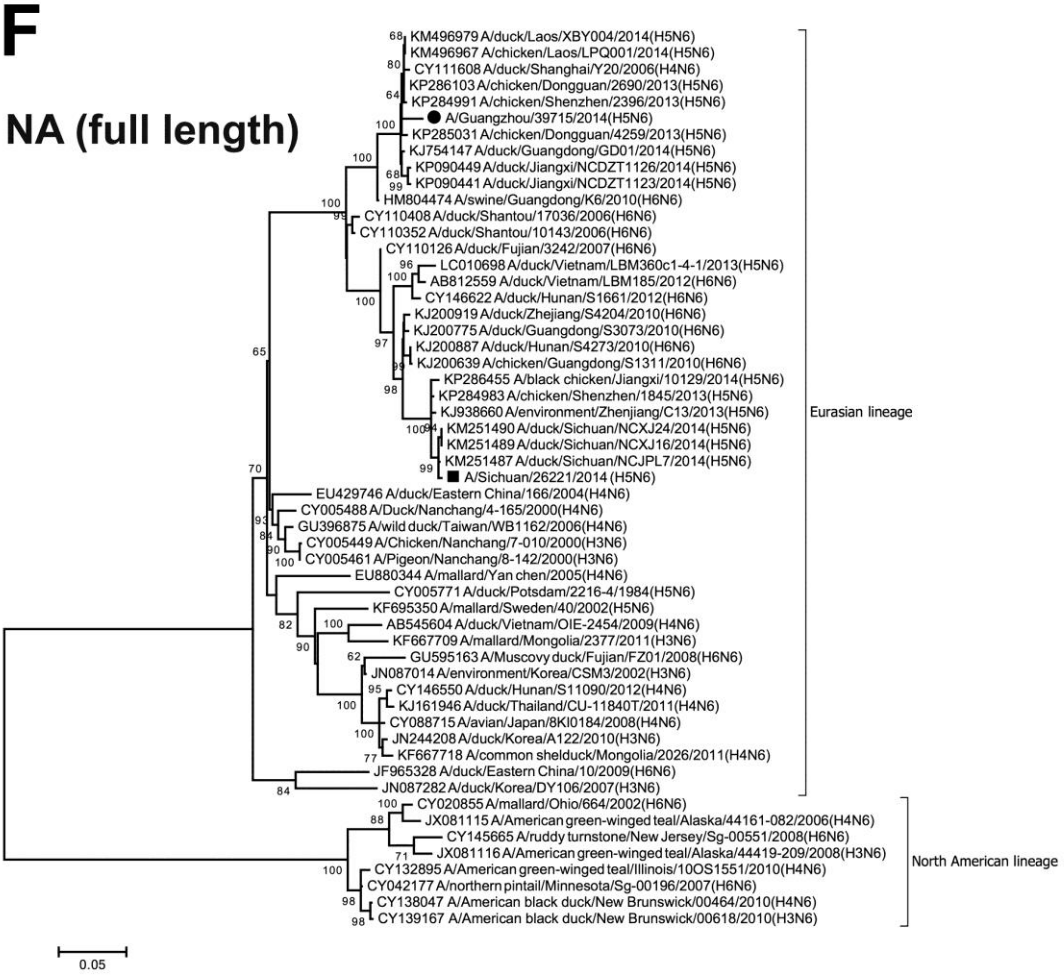

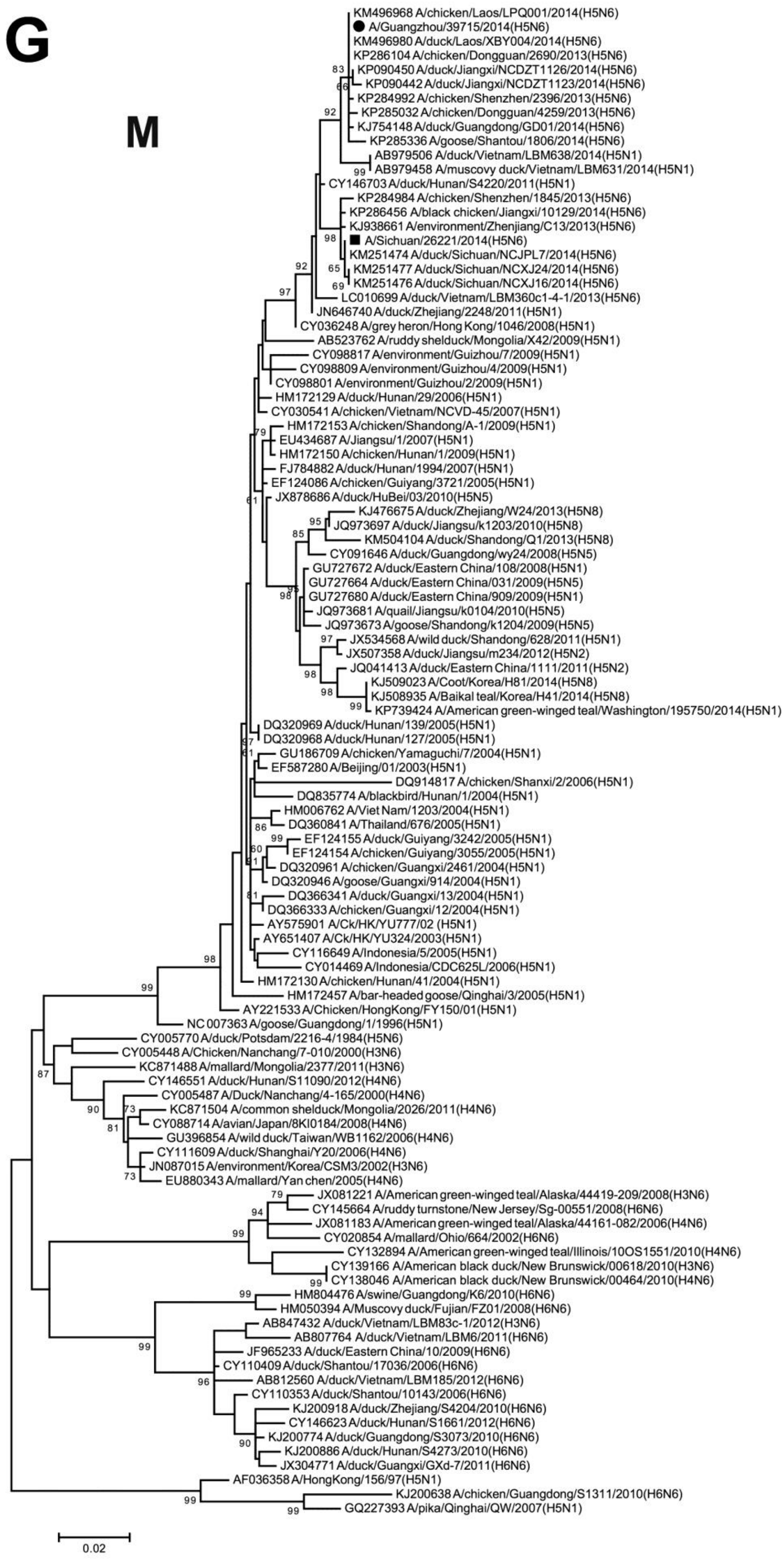
G
M
KM496968 A/chicken/Laos/LPQ001/2014(H5N6)
A/Guangzhou/39715/2014(H5N6)
KM496980 A/duck/Laos/XBY004/2014(H5N6)
KP286104 A/chicken/Dongguan/2690/2013(H5N6)
KP090450 A/duck/Jiangxi/NCDZT1126/2014(H5N6)
KP090442 A/duck/Jiangxi/NCDZT1123/2014(H5N6)
KP284992 A/chicken/Shenzhen/2396/2013(H5N6)
KP285032 A/chicken/Dongguan/4259/2013(H5N6)
KJ754148 A/duck/Guangdong/GD01/2014(H5N6)
KP285336 A/goose/Shantou/1806/2014(H5N6)
AB979506 A/duck/Vietnam/LBM638/2014(H5N1)
AB979458 A/muscovy duck/Vietnam/LBM631/2014(H5N1)
CY146703 A/duck/Hunan/S4220/2011(H5N1)
KP284984 A/chicken/Shenzhen/1845/2013(H5N6)
KP286456 A/black chicken/Jiangxi/10129/2014(H5N6)
KJ938661 A/environment/Zhenjiang/C13/2013(H5N6)
A/Sichuan/26221/2014(H5N6)
KM251474 A/duck/Sichuan/NCJPL7/2014(H5N6)
KM251477 A/duck/Sichuan/NCXJ24/2014(H5N6)
KM251476 A/duck/Sichuan/NCXJ16/2014(H5N6)
LC010699 A/duck/Vietnam/LBM360c1-4-1/2013(H5N6)
JN646740 A/duck/Zhejiang/2248/2011(H5N1)
CY036248 A/grey heron/Hong Kong/1046/2008(H5N1)
AB523762 A/ruddy shelduck/Mongolia/X42/2009(H5N1)
CY098817 A/environment/Guizhou/7/2009(H5N1)
CY098809 A/environment/Guizhou/4/2009(H5N1)
CY098801 A/environment/Guizhou/2/2009(H5N1)
HM172129 A/duck/Hunan/29/2006(H5N1)
CY030541 A/chicken/Vietnam/NCVD-45/2007(H5N1)
HM172153 A/chicken/Shandong/A-1/2009(H5N1)
EU434687 A/Jiangsu/1/2007(H5N1)
HM172150 A/chicken/Hunan/1/2009(H5N1)
FJ784882 A/duck/Hunan/1994/2007(H5N1)
EF124086 A/chicken/Guiyang/3721/2005(H5N1)
JX878686 A/duck/HuBei/03/2010(H5N5)
KJ476675 A/duck/Zhejiang/W24/2013(H5N8)
JQ973697 A/duck/Jiangsu/k1203/2010(H5N8)
KM504104 A/duck/Shandong/Q1/2013(H5N8)
CY091646 A/duck/Guangdong/wy24/2008(H5N5)
GU727672 A/duck/Eastern China/108/2008(H5N1)
GU727664 A/duck/Eastern China/031/2009(H5N5)
GU727680 A/duck/Eastern China/909/2009(H5N1)
JQ973681 A/quail/Jiangsu/k0104/2010(H5N5)
JQ973673 A/goose/Shandong/k1204/2009(H5N5)
JX534568 A/wild duck/Shandong/628/2011(H5N1)
JX507358 A/duck/Jiangsu/m234/2012(H5N2)
JQ041413 A/duck/Eastern China/1111/2011(H5N2)
KJ509023 A/Coot/Korea/H81/2014(H5N8)
KJ508935 A/Baikal teal/Korea/H41/2014(H5N8)
KP739424 A/American green-winged teal/Washington/195750/2014(H5N1)
DQ320969 A/duck/Hunan/139/2005(H5N1)
DQ320968 A/duck/Hunan/127/2005(H5N1)
GU186709 A/chicken/Yamaguchi/7/2004(H5N1)
EF587280 A/Beijing/01/2003(H5N1)
DQ914817 A/chicken/Shanxi/2/2006(H5N1)
DQ835774 A/blackbird/Hunan/1/2004(H5N1)
HM006762 A/Viet Nam/1203/2004(H5N1)
DQ360841 A/Thailand/676/2005(H5N1)
EF124155 A/duck/Guiyang/3242/2005(H5N1)
EF124154 A/chicken/Guiyang/3055/2005(H5N1)
DQ320961 A/chicken/Guangxi/2461/2004(H5N1)
DQ320946 A/goose/Guangxi/914/2004(H5N1)
DQ366341 A/duck/Guangxi/13/2004(H5N1)
DQ366333 A/chicken/Guangxi/12/2004(H5N1)
AY575901 A/Ck/HK/YU777/02 (H5N1)
AY651407 A/Ck/HK/YU324/2003(H5N1)
CY116649 A/Indonesia/5/2005(H5N1)
CY014469 A/Indonesia/CDC625L/2006(H5N1)
HM172130 A/chicken/Hunan/41/2004(H5N1)
HM172457 A/bar-headed goose/Qinghai/3/2005(H5N1)
AY221533 A/Chicken/HongKong/FY150/01(H5N1)
NC 007363 A/goose/Guangdong/1/1996(H5N1)
CY005770 A/duck/Potsdam/2216-4/1984(H5N6)
CY005448 A/Chicken/Nanchang/7-010/2000(H3N6)
KC871488 A/mallard/Mongolia/2377/2011(H3N6)
CY146551 A/duck/Hunan/S11090/2012(H4N6)
CY005487 A/Duck/Nanchang/4-165/2000(H4N6)
KC871504 A/common shelduck/Mongolia/2026/2011(H4N6)
CY088714 A/avian/Japan/8KI0184/2008(H4N6)
GU396854 A/wild duck/Taiwan/WB1162/2006(H4N6)
CY111609 A/duck/Shanghai/Y20/2006(H4N6)
JN087015 A/environment/Korea/CSM3/2002(H3N6)
EU880343 A/mallard/Yan chen/2005(H4N6)
JX081221 A/American green-winged teal/Alaska/44419-209/2008(H3N6)
CY145664 A/ruddy turnstone/New Jersey/Sg-00551/2008(H6N6)
JX081183 A/American green-winged teal/Alaska/44161-082/2006(H4N6)
CY020854 A/mallard/Ohio/664/2002(H6N6)
CY132894 A/American green-winged teal/Illinois/10OS1551/2010(H4N6)
CY139166 A/American black duck/New Brunswick/00618/2010(H3N6)
CY138046 A/American black duck/New Brunswick/00464/2010(H4N6)
HM804476 A/swine/Guangdong/K6/2010(H6N6)
HM050394 A/Muscovy duck/Fujian/FZ01/2008(H6N6)
AB847432 A/duck/Vietnam/LBM83c-1/2012(H3N6)
AB807764 A/duck/Vietnam/LBM6/2011(H6N6)
JF965233 A/duck/Eastern China/10/2009(H6N6)
CY110409 A/duck/Shantou/17036/2006(H6N6)
AB812560 A/duck/Vietnam/LBM185/2012(H6N6)
CY110353 A/duck/Shantou/10143/2006(H6N6)
KJ200918 A/duck/Zhejiang/S4204/2010(H6N6)
CY146623 A/duck/Hunan/S1661/2012(H6N6)
KJ200774 A/duck/Guangdong/S3073/2010(H6N6)
KJ200886 A/duck/Hunan/S4273/2010(H6N6)
JX304771 A/duck/Guangxi/GXd-7/2011(H6N6)
AF036358 A/HongKong/156/97(H5N1)
KJ200638 A/chicken/Guangdong/S1311/2010(H6N6)
GQ227393 A/pika/Qinghai/QW/2007(H5N1)
0.02

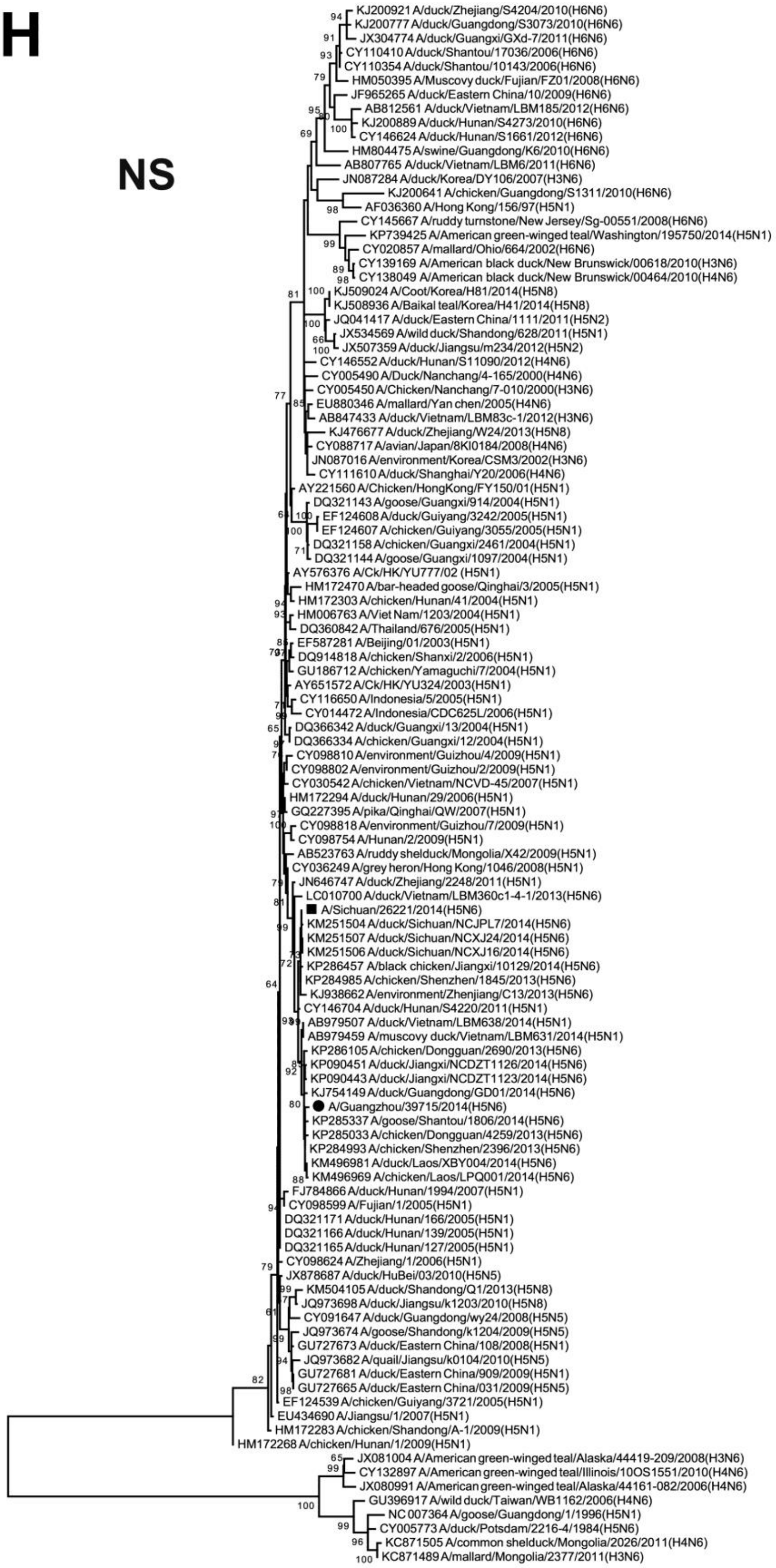

Full phylogenetic trees of polymerase basic (PB2) (Technical Appendix Figure 1A), PB1 (Technical Appendix Figure 1B), polymerase acidic (PA) (Technical Appendix

Figure 1C), hemagglutinin (HA) (Technical Appendix Figure 1D), nucleoprotein (NP) (Technical Appendix Figure 1E), neuraminidase (NA) (Technical Appendix Figure 1F), M (Technical Appendix Figure 1G), and nonstructural (NS) (Technical Appendix Figure 1H) genes. Maximum-likelihood trees were constructed by using the general time reversible + gama distribution + invariant sites (GTR + Γ + I) model in MEGA 6.06 (http://www.megasoftware.net). Bootstrap values were calculated on 1,000 replicates; only values >60% are shown. A/Guangzhou/39715/2014 and A/Sichuan/26221/2014 are marked by a circle and a square, respectively. Scale bars indicate nucleotide substitutions per site.
